# Notes and Notices

**Published:** 1887-11

**Authors:** 


### NOTES AND NOTICES.

Dr. Emma T. Schreiner has established an office at 123 West Chelten Avenue, Germantown, Philadelphia.

For Sale.—A rare chance to purchase a fine portrait of Hahnemann, by Huntington, is now offered. For particulars, apply at this office.

